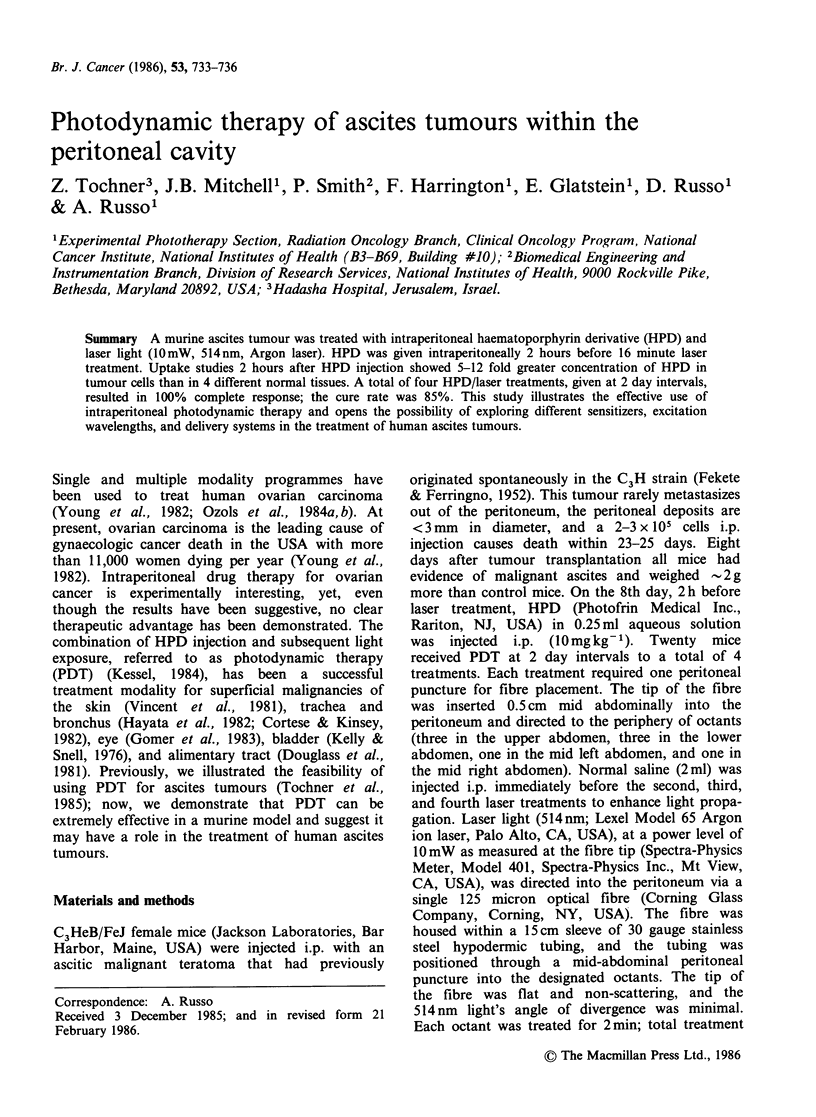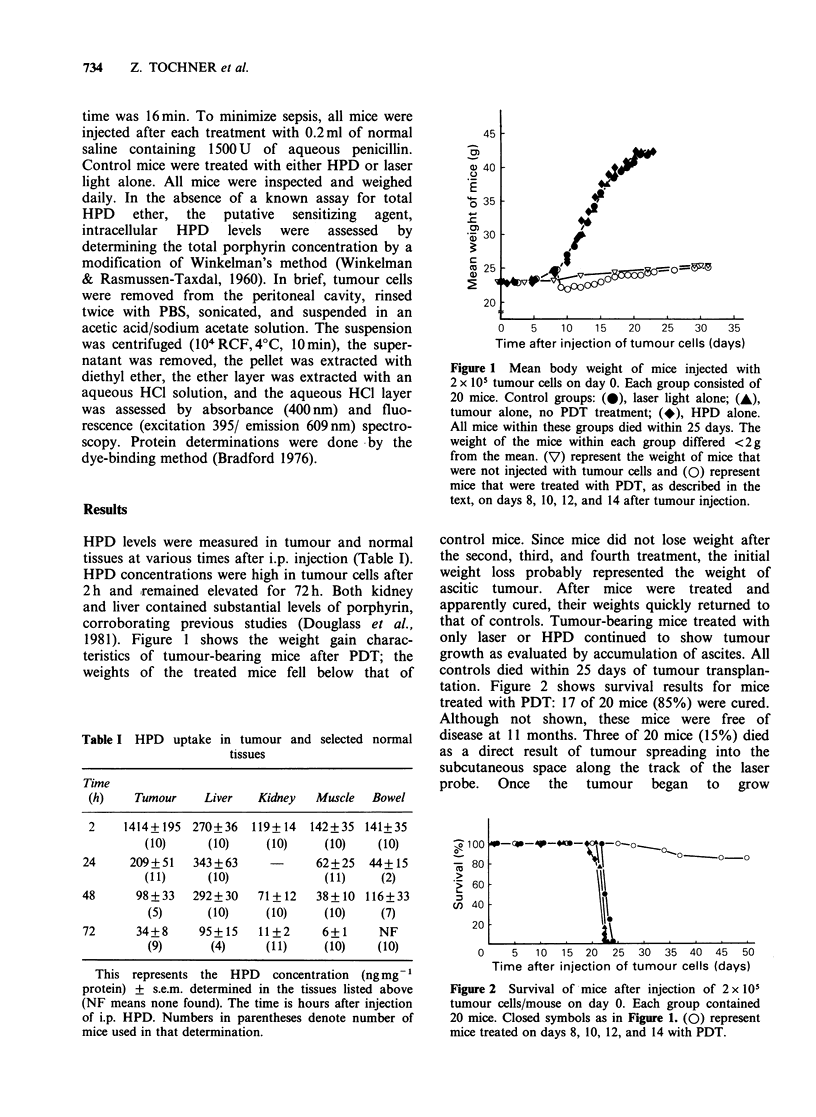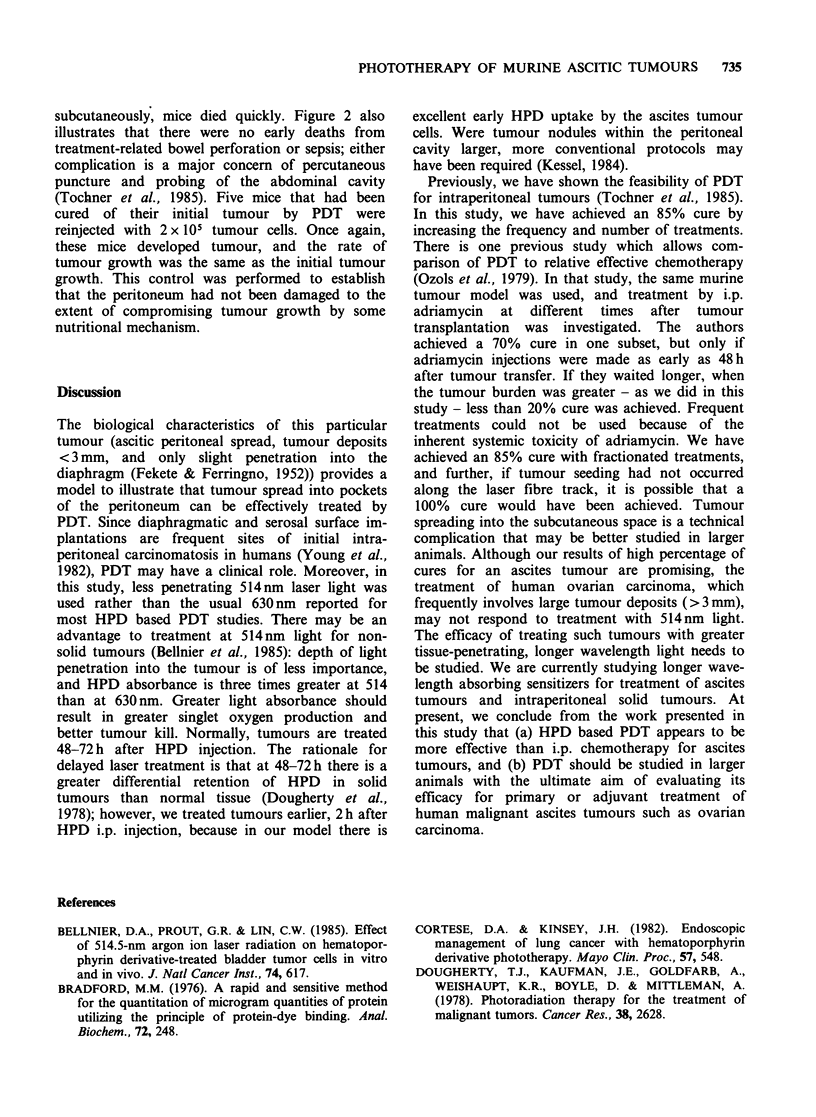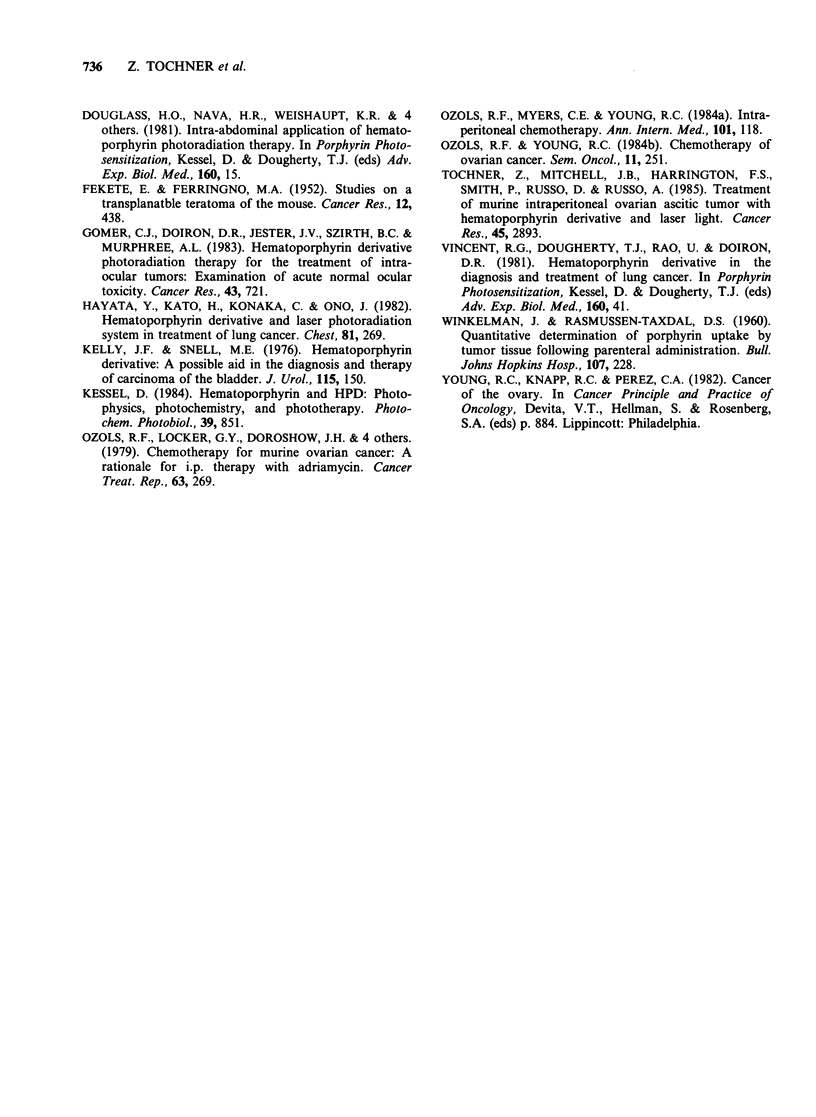# Photodynamic therapy of ascites tumours within the peritoneal cavity.

**DOI:** 10.1038/bjc.1986.126

**Published:** 1986-06

**Authors:** Z. Tochner, J. B. Mitchell, P. Smith, F. Harrington, E. Glatstein, D. Russo, A. Russo

## Abstract

A murine ascites tumour was treated with intraperitoneal haematoporphyrin derivative (HPD) and laser light (10mW, 514nm, Argon laser). HPD was given intraperitoneally 2 hours before 16 minute laser treatment. Uptake studies 2 hours after HPD injection showed 5-12 fold greater concentration of HPD in tumour cells than in 4 different normal tissues. A total of four HPD/laser treatments, given at 2 day intervals, resulted in 100% complete response; the cure rate was 85%. This study illustrates the effective use of intraperitoneal photodynamic therapy and opens the possibility of exploring different sensitizers, excitation wavelengths, and delivery systems in the treatment of human ascites tumours.


*Br. J. Cancer* (1986), **53**, 733–736

### Photodynamic therapy of ascites tumours within the peritoneal cavity

Z. Tochner[3], J.B. Mitchell[1], P. Smith[2], F. Harrington[1], E. Glatstein[1], D. Russo[1] & A. Russo[1]

[1]*Experimental Phototherapy Section, Radiation Oncology Branch, Clinical Oncology Program, National Cancer Institute, National Institutes of Health (B3–B69, Building #10);* [2]*Biomedical Engineering and Instrumentation Branch, Division of Research Services, National Institutes of Health, 9000 Rockville Pike, Bethesda, Maryland 20892, USA;* [3]*Hadasha Hospital, Jerusalem, Israel.*

**Summary** A murine ascites tumour was treated with intraperitoneal haematoporphyrin derivative (HPD) and laser light (10 mW, 514 nm, Argon laser). HPD was given intraperitoneally 2 hours before 16 minute laser treatment. Uptake studies 2 hours after HPD injection showed 5–12 fold greater concentration of HPD in tumour cells than in 4 different normal tissues. A total of four HPD/laser treatments, given at 2 day intervals, resulted in 100% complete response; the cure rate was 85%. This study illustrates the effective use of intraperitoneal photodynamic therapy and opens the possibility of exploring different sensitizers, excitation wavelengths, and delivery systems in the treatment of human ascites tumours.

Single and multiple modality programmes have been used to treat human ovarian carcinoma (Young *et al.*, 1982; Ozols *et al.*, 1984*a,b*). At present, ovarian carcinoma is the leading cause of gynaecologic cancer death in the USA with more than 11,000 women dying per year (Young *et al.*, 1982). Intraperitoneal drug therapy for ovarian cancer is experimentally interesting, yet, even though the results have been suggestive, no clear therapeutic advantage has been demonstrated. The combination of HPD injection and subsequent light exposure, referred to as photodynamic therapy (PDT) (Kessel, 1984), has been a successful treatment modality for superficial malignancies of the skin (Vincent *et al.*, 1981), trachea and bronchus (Hayata *et al.*, 1982; Cortese & Kinsey, 1982), eye (Gomer *et al.*, 1983), bladder (Kelly & Snell, 1976), and alimentary tract (Douglass *et al.*, 1981). Previously, we illustrated the feasibility of using PDT for ascites tumours (Tochner *et al.*, 1985); now, we demonstrate that PDT can be extremely effective in a murine model and suggest it may have a role in the treatment of human ascites tumours.

### Materials and methods

$C_3$HeB/FeJ female mice (Jackson Laboratories, Bar Harbor, Maine, USA) were injected i.p. with an ascitic malignant teratoma that had previously originated spontaneously in the $C_3$H strain (Fekete & Ferringno, 1952). This tumour rarely metastasizes out of the peritoneum, the peritoneal deposits are <3 mm in diameter, and a $2–3 \times 10^5$ cells i.p. injection causes death within 23–25 days. Eight days after tumour transplantation all mice had evidence of malignant ascites and weighed ~2 g more than control mice. On the 8th day, 2 h before laser treatment, HPD (Photofrin Medical Inc., Rariton, NJ, USA) in 0.25 ml aqueous solution was injected i.p. ($10\,mg\,kg^{-1}$). Twenty mice received PDT at 2 day intervals to a total of 4 treatments. Each treatment required one peritoneal puncture for fibre placement. The tip of the fibre was inserted 0.5 cm mid abdominally into the peritoneum and directed to the periphery of octants (three in the upper abdomen, three in the lower abdomen, one in the mid left abdomen, and one in the mid right abdomen). Normal saline (2 ml) was injected i.p. immediately before the second, third, and fourth laser treatments to enhance light propagation. Laser light (514 nm; Lexel Model 65 Argon ion laser, Palo Alto, CA, USA), at a power level of 10 mW as measured at the fibre tip (Spectra-Physics Meter, Model 401, Spectra-Physics Inc., Mt View, CA, USA), was directed into the peritoneum via a single 125 micron optical fibre (Corning Glass Company, Corning, NY, USA). The fibre was housed within a 15 cm sleeve of 30 gauge stainless steel hypodermic tubing, and the tubing was positioned through a mid-abdominal peritoneal puncture into the designated octants. The tip of the fibre was flat and non-scattering, and the 514 nm light's angle of divergence was minimal. Each octant was treated for 2 min; total treatment

Correspondence: A. Russo
Received 3 December 1985; and in revised form 21 February 1986.

© The Macmillan Press Ltd., 1986

time was 16 min. To minimize sepsis, all mice were injected after each treatment with 0.2 ml of normal saline containing 1500 U of aqueous penicillin. Control mice were treated with either HPD or laser light alone. All mice were inspected and weighed daily. In the absence of a known assay for total HPD ether, the putative sensitizing agent, intracellular HPD levels were assessed by determining the total porphyrin concentration by a modification of Winkelman's method (Winkelman & Rasmussen-Taxdal, 1960). In brief, tumour cells were removed from the peritoneal cavity, rinsed twice with PBS, sonicated, and suspended in an acetic acid/sodium acetate solution. The suspension was centrifuged ($10^4$ RCF, 4°C, 10 min), the supernatant was removed, the pellet was extracted with diethyl ether, the ether layer was extracted with an aqueous HCl solution, and the aqueous HCl layer was assessed by absorbance (400 nm) and fluorescence (excitation 395/ emission 609 nm) spectroscopy. Protein determinations were done by the dye-binding method (Bradford 1976).

### Results

HPD levels were measured in tumour and normal tissues at various times after i.p. injection (Table I). HPD concentrations were high in tumour cells after 2 h and remained elevated for 72 h. Both kidney and liver contained substantial levels of porphyrin, corroborating previous studies (Douglass *et al.*, 1981). Figure 1 shows the weight gain characteristics of tumour-bearing mice after PDT; the weights of the treated mice fell below that of control mice. Since mice did not lose weight after the second, third, and fourth treatment, the initial weight loss probably represented the weight of ascitic tumour. After mice were treated and apparently cured, their weights quickly returned to that of controls. Tumour-bearing mice treated with only laser or HPD continued to show tumour growth as evaluated by accumulation of ascites. All controls died within 25 days of tumour transplantation. Figure 2 shows survival results for mice treated with PDT: 17 of 20 mice (85%) were cured. Although not shown, these mice were free of disease at 11 months. Three of 20 mice (15%) died as a direct result of tumour spreading into the subcutaneous space along the track of the laser probe. Once the tumour began to grow

**Table I** HPD uptake in tumour and selected normal tissues

| *Time (h)* | *Tumour* | *Liver* | *Kidney* | *Muscle* | *Bowel* |
|---|---|---|---|---|---|
| 2 | 1414±195 (10) | 270±36 (10) | 119±14 (10) | 142±35 (10) | 141±35 (10) |
| 24 | 209±51 (11) | 343±63 (10) | — | 62±25 (11) | 44±15 (2) |
| 48 | 98±33 (5) | 292±30 (10) | 71±12 (10) | 38±10 (10) | 116±33 (7) |
| 72 | 34±8 (9) | 95±15 (4) | 11±2 (11) | 6±1 (10) | NF (10) |

This represents the HPD concentration ($ng\,mg^{-1}$ protein) ± s.e.m. determined in the tissues listed above (NF means none found). The time is hours after injection of i.p. HPD. Numbers in parentheses denote number of mice used in that determination.

**Figure 1** Mean body weight of mice injected with $2\times10^5$ tumour cells on day 0. Each group consisted of 20 mice. Control groups: (●), laser light alone; (▲), tumour alone, no PDT treatment; (◆), HPD alone. All mice within these groups died within 25 days. The weight of the mice within each group differed <2 g from the mean. (▽) represent the weight of mice that were not injected with tumour cells and (○) represent mice that were treated with PDT, as described in the text, on days 8, 10, 12, and 14 after tumour injection.

**Figure 2** Survival of mice after injection of $2\times10^5$ tumour cells/mouse on day 0. Each group contained 20 mice. Closed symbols as in **Figure 1.** (○) represent mice treated on days 8, 10, 12, and 14 with PDT.

subcutaneously, mice died quickly. Figure 2 also illustrates that there were no early deaths from treatment-related bowel perforation or sepsis; either complication is a major concern of percutaneous puncture and probing of the abdominal cavity (Tochner *et al.*, 1985). Five mice that had been cured of their initial tumour by PDT were reinjected with $2 \times 10^5$ tumour cells. Once again, these mice developed tumour, and the rate of tumour growth was the same as the initial tumour growth. This control was performed to establish that the peritoneum had not been damaged to the extent of compromising tumour growth by some nutritional mechanism.

### Discussion

The biological characteristics of this particular tumour (ascitic peritoneal spread, tumour deposits <3 mm, and only slight penetration into the diaphragm (Fekete & Ferringno, 1952)) provides a model to illustrate that tumour spread into pockets of the peritoneum can be effectively treated by PDT. Since diaphragmatic and serosal surface implantations are frequent sites of initial intraperitoneal carcinomatosis in humans (Young *et al.*, 1982), PDT may have a clinical role. Moreover, in this study, less penetrating 514 nm laser light was used rather than the usual 630 nm reported for most HPD based PDT studies. There may be an advantage to treatment at 514 nm light for non-solid tumours (Bellnier *et al.*, 1985): depth of light penetration into the tumour is of less importance, and HPD absorbance is three times greater at 514 than at 630 nm. Greater light absorbance should result in greater singlet oxygen production and better tumour kill. Normally, tumours are treated 48–72 h after HPD injection. The rationale for delayed laser treatment is that at 48–72 h there is a greater differential retention of HPD in solid tumours than normal tissue (Dougherty *et al.*, 1978); however, we treated tumours earlier, 2 h after HPD i.p. injection, because in our model there is excellent early HPD uptake by the ascites tumour cells. Were tumour nodules within the peritoneal cavity larger, more conventional protocols may have been required (Kessel, 1984).

Previously, we have shown the feasibility of PDT for intraperitoneal tumours (Tochner *et al.*, 1985). In this study, we have achieved an 85% cure by increasing the frequency and number of treatments. There is one previous study which allows comparison of PDT to relative effective chemotherapy (Ozols *et al.*, 1979). In that study, the same murine tumour model was used, and treatment by i.p. adriamycin at different times after tumour transplantation was investigated. The authors achieved a 70% cure in one subset, but only if adriamycin injections were made as early as 48 h after tumour transfer. If they waited longer, when the tumour burden was greater – as we did in this study – less than 20% cure was achieved. Frequent treatments could not be used because of the inherent systemic toxicity of adriamycin. We have achieved an 85% cure with fractionated treatments, and further, if tumour seeding had not occurred along the laser fibre track, it is possible that a 100% cure would have been achieved. Tumour spreading into the subcutaneous space is a technical complication that may be better studied in larger animals. Although our results of high percentage of cures for an ascites tumour are promising, the treatment of human ovarian carcinoma, which frequently involves large tumour deposits (>3 mm), may not respond to treatment with 514 nm light. The efficacy of treating such tumours with greater tissue-penetrating, longer wavelength light needs to be studied. We are currently studying longer wavelength absorbing sensitizers for treatment of ascites tumours and intraperitoneal solid tumours. At present, we conclude from the work presented in this study that (a) HPD based PDT appears to be more effective than i.p. chemotherapy for ascites tumours, and (b) PDT should be studied in larger animals with the ultimate aim of evaluating its efficacy for primary or adjuvant treatment of human malignant ascites tumours such as ovarian carcinoma.

### References

BELLNIER, D.A., PROUT, G.R. & LIN, C.W. (1985). Effect of 514.5-nm argon ion laser radiation on hematoporphyrin derivative-treated bladder tumor cells in vitro and in vivo. *J. Natl Cancer Inst.*, **74**, 617.

BRADFORD, M.M. (1976). A rapid and sensitive method for the quantitation of microgram quantities of protein utilizing the principle of protein-dye binding. *Anal. Biochem.*, **72**, 248.

CORTESE, D.A. & KINSEY, J.H. (1982). Endoscopic management of lung cancer with hematoporphyrin derivative phototherapy. *Mayo Clin. Proc.*, **57**, 548.

DOUGHERTY, T.J., KAUFMAN, J.E., GOLDFARB, A., WEISHAUPT, K.R., BOYLE, D. & MITTLEMAN, A. (1978). Photoradiation therapy for the treatment of malignant tumors. *Cancer Res.*, **38**, 2628.

DOUGLASS, H.O., NAVA, H.R., WEISHAUPT, K.R. & 4 others. (1981). Intra-abdominal application of hematoporphyrin photoradiation therapy. In *Porphyrin Photosensitization*, Kessel, D. & Dougherty, T.J. (eds) *Adv. Exp. Biol. Med.*, **160**, 15.

FEKETE, E. & FERRINGNO, M.A. (1952). Studies on a transplanatble teratoma of the mouse. *Cancer Res.*, **12**, 438.

GOMER, C.J., DOIRON, D.R., JESTER, J.V., SZIRTH, B.C. & MURPHREE, A.L. (1983). Hematoporphyrin derivative photoradiation therapy for the treatment of intraocular tumors: Examination of acute normal ocular toxicity. *Cancer Res.*, **43**, 721.

HAYATA, Y., KATO, H., KONAKA, C. & ONO, J. (1982). Hematoporphyrin derivative and laser photoradiation system in treatment of lung cancer. *Chest*, **81**, 269.

KELLY, J.F. & SNELL, M.E. (1976). Hematoporphyrin derivative: A possible aid in the diagnosis and therapy of carcinoma of the bladder. *J. Urol.*, **115**, 150.

KESSEL, D. (1984). Hematoporphyrin and HPD: Photophysics, photochemistry, and phototherapy. *Photochem. Photobiol.*, **39**, 851.

OZOLS, R.F., LOCKER, G.Y., DOROSHOW, J.H. & 4 others. (1979). Chemotherapy for murine ovarian cancer: A rationale for i.p. therapy with adriamycin. *Cancer Treat. Rep.*, **63**, 269.

OZOLS, R.F., MYERS, C.E. & YOUNG, R.C. (1984a). Intraperitoneal chemotherapy. *Ann. Intern. Med.*, **101**, 118.

OZOLS, R.F. & YOUNG, R.C. (1984b). Chemotherapy of ovarian cancer. *Sem. Oncol.*, **11**, 251.

TOCHNER, Z., MITCHELL, J.B., HARRINGTON, F.S., SMITH, P., RUSSO, D. & RUSSO, A. (1985). Treatment of murine intraperitoneal ovarian ascitic tumor with hematoporphyrin derivative and laser light. *Cancer Res.*, **45**, 2893.

VINCENT, R.G., DOUGHERTY, T.J., RAO, U. & DOIRON, D.R. (1981). Hematoporphyrin derivative in the diagnosis and treatment of lung cancer. In *Porphyrin Photosensitization*, Kessel, D. & Dougherty, T.J. (eds) *Adv. Exp. Biol. Med.*, **160**, 41.

WINKELMAN, J. & RASMUSSEN-TAXDAL, D.S. (1960). Quantitative determination of porphyrin uptake by tumor tissue following parenteral administration. *Bull. Johns Hopkins Hosp.*, **107**, 228.

YOUNG, R.C., KNAPP, R.C. & PEREZ, C.A. (1982). Cancer of the ovary. In *Cancer Principle and Practice of Oncology*, Devita, V.T., Hellman, S. & Rosenberg, S.A. (eds) p. 884. Lippincott: Philadelphia.